# Research on the Influencing Factors of Audience Popularity Level of COVID-19 Videos during the COVID-19 Pandemic

**DOI:** 10.3390/healthcare9091159

**Published:** 2021-09-04

**Authors:** Jingfang Liu, Caiying Lu, Shuangjinhua Lu

**Affiliations:** School of Management, Shanghai University, Shanghai 200444, China; jingfangliu2014@hotmail.com (J.L.); lushuangjinhua@shu.edu.cn (S.L.)

**Keywords:** COVID-19, information adoption model, text analysis, public health emergency

## Abstract

(1) Background: During the COVID-19 pandemic, users share and obtain COVID-19 information through video platforms, but only a few COVID-19 videos become popular among most audiences. Therefore, it is a very interesting and important research question to explore the influencing factors of the popularity of COVID-19 videos during the COVID-19 pandemic; (2) Our research collects video data related to the keyword “COVID-19” on video platform, the data are analyzed by content analysis and empirical analysis. We then constructed a theoretical model based on the information adoption model; (3) A total of 251 videos were divided into three categories. The least common category was the data and analysis category (11.2%), followed by the prevention and control status category (13.5%); the knowledge and general science category was the most common (75.3%). From the perspective of video quality, the information sources of most videos are relatively reliable, and the content of medical information is low. The research results showed that short video lengths, longer descriptions, more reliable video sources and lower medical information content were more popular with audiences. Audiences are more likely to be attracted to videos in the prevention and control status category and knowledge and general science category. Videos uploaded by uploaders who have a higher influence are more popular with audiences; (4) Conclusions: During the COVID-19 pandemic, information quality (video length, description length, video content type, and medical information and content index) and source credibility (information source reliability, influence and certification type) all significantly influence the popularity level of COVID-19 videos. Our research conclusions can provide management suggestions for the platform, make videos released by uploaders more popular with audiences, and help audiences better understand COVID-19 information and make prevention and control efforts.

## 1. Introduction

In December 2019, a new type of coronavirus [1,2], which can cause respiratory disease, was discovered. On 30 January 2020, the World Health Organization declared this outbreak a public health emergency of international concern [3], and named the disease “Coronavirus Disease 2019” (COVID-19) on 11 February [4]. COVID-19 has rapidly spread across the globe, and the pandemic has wreaked havoc not only on public health and the economy [5,6,7], but on all aspects of society. In terms of education, screen-mediated online education has become the mainstream during the epidemic [8], forcing urgent changes in education methods and posing new challenges to educators [9,10]. At the same time, the epidemic has greatly changed people’s lifestyle, resulting in more negative emotions, such as anger and anxiety, and the topic of discussion has also turned to issues related to biology and death [11]. In the face of the powerful destructive power and unknowns of COVID-19, the general public is in high panic, complicating the spread of the disease [12]. Therefore, people hope to alleviate this sentiment by learning more information.

During the pandemic, social media became the main source of information for people to understand it [13]. A UNESCO study of 1735 Gen Z in more than 100 countries showed that although more than half of Gen Z worried about Internet security, but only 22% of respondents said they have never shared their private information on the Internet [14]. The biggest feature of Gen Z (people born in 1990–2009) is that practically no individual in this generation is unfamiliar with the Internet, and the Internet is involved in their lives [15]. They are digital aborigines who know little about the era without social media and Internet [16]. Privacy issues has been paid more attention by people in the process of information and knowledge digitization. Gen Z, who grew up in the digital era, do not pay enough attention to privacy because they have not experienced the process of change. Compared with other generations, Gen Z can easily obtain knowledge and information from different information sources on the Internet by mastering professional technologies [17] in China, Gen Z is also the main force of the Internet. CNNIC’s survey report has shown that the number of Internet users in China is 989 million, among which Gen Z Internet users account for 31.3% [18], thus becoming the main force of the Internet. Gen Z, as active Internet user, who is more willing to share publicly and complete the acquisition and sharing of knowledge and information on the Internet.

In a fast-paced environment, short attention and distraction have become the generational characteristics of Gen Z [19]. Rothman’s research shows that Gen Z respond more strongly to information presented in visual form, but have a shorter attention span [20]. The video platform caters to the needs of “Generation Z” Internet users, limits the video duration, and adopts intelligent recommendation algorithms to continuously recommend new videos in a personalized manner [21]. QuestMobile’s survey report, Gen Z’s interest preference for videos is 78.1% [22]. Therefore, video is the form of information favored by contemporary mobile Internet users, and research on video has become an indispensable part of the research on Internet information transmission.

With the spread of the epidemic, the number of infections and deaths continues to increase. Videos about COVID-19 on various video platforms have exploded, and people hope to obtain and share information about COVID-19 through videos. When such explosive information appears in front of an audience, the audience will have more choices, but only a few videos can gain major popularity [23]. Every uploader wants his or her video to be popular, so how to make the video stand out from the numerous videos has become an issue of concern for uploaders. Therefore, it is a very interesting and important research question to explore the influencing factors of the popularity of videos in the context of COVID-19.

Several obvious gaps in the current study could be narrowed. First of all, studies on the transmission of text information have used a variety of different platforms as data sources, including Twitter [24,25], Weibo [26], and Facebook [27]. However, most research on epidemic information spread in the form of video has used the YouTube platform as the information source [28,29,30,31]. Compared with research on traditional text, research on videos tends to focus on a relatively single platform; there are many other emerging video platforms worth studying. In addition, Chinese users mostly use domestic video platforms for information related to COVID-19 and social communication. The study of Video platforms in China can well fill in data gap of YouTube.

Secondly, previous studies on the factors affecting the popularity of COVID-19 videos have mainly been conducted from the perspective of certain video features [28,30], while few studies have comprehensively considered various indicators of video features. In addition, few studies have considered the effects of uploader features on video popularity. In this paper, uploader features are innovatively incorporated into the model, when the independent variable index is constructed.

According to information adoption model, independent variables including information quality variables (video length, description length, content category, and medical information and content index (MICI)) and source credibility variables (information source reliability (modified DISCERN), influence, and certification type) are established, and a text analysis method is used to study the content category of the videos. The modified DISCERN and MICI variables are quantitatively analyzed through the content analysis method, while information quality and source credibility are included in the regression model.

## 2. Theoretical Background and Hypothesis

### 2.1. Theoretical Background

The information adoption model (IAM) combines the technology acceptance model [32] and the dual-process model of information influence [33,34]. It has been widely used in the information system field, in order to explain the process of information processing [35]. IAM interprets how the central and peripheral cues of information affect the behavior of information adoption, by taking the intentions of information perceived by the information receiver as the intermediary [36]. Central cues are generated when people think about information related to the problem. The quality of information (relevance, completeness, accuracy, and timeliness) significantly affects the receiver’s information adoption on the center line [36,37]. Peripheral cues are composed of simple cues, such as the credibility of information sources [38]. When considering an Internet platform, IAM has been widely used to explain the process of information adoption in the platform [39].

### 2.2. Hypothesis

#### 2.2.1. Information Quality

The length of a video refers to the playing time of a video, which is an important index when studying the factors influencing the popularity of a video. The public usually uses fragmented time to watch videos on mobile devices, such that shorter videos are more in line with public consumption habits [40]. Guo (2014) found that shorter videos may be of higher quality [41], due to the limitation of video length, which requires more careful planning of video content arrangement. So, we obtain the first hypothesis:

**Hypothesis** **1** **(H1).** *During the COVID-19 pandemic, the shorter the video length, the more popular the COVID-19 video is.*

The length of the video title is the number of characters in the video title. During the COVID-19 pandemic, the public has come into a state of panic, hoping to use video platforms to obtain information in order to alleviate their anxiety, with a strong purpose. video platform has a word limit for video title display; if the title is too long, the integrity of information and perceived usefulness of the video will be affected.The video information may not be adopted and the video can become unpopular with audiences [42,43].

**Hypothesis** **2** **(H2).** *During the COVID-19 pandemic, the shorter the video title, the more popular the COVID-19 video is.*

The description length of a video refers to the length of the description text of the video. The description can help the audience to understand the video content, and is an extension of the video title. Thackeray (2013) has shown that the more descriptive information a video contains, the more views it receives [44]. The longer the video description, the more textual information it contains, and the more complete the information. Thus, audiences find the information useful and attractive.

**Hypothesis** **3** **(H3).** *During the COVID-19 pandemic, the longer the video description, the more popular the COVID-19 video will be.*

Video content type refers to the type of information contained in the video, which requires qualitative analysis using text analysis. Uncertainty reduction theory states that, when a crisis occurs, people tend to learn more objective details related to the crisis, in order to reduce the associated uncertainty [45]. Due to different goals, individuals have different needs when obtaining information, so they selectively adopt the information they perceive to be useful [46]. The perceived usefulness of COVID-19 videos with different content is different, which is reflected in the difference in their popularity [27]. Therefore, the content type of the video is related to the perceived usefulness of the information.

**Hypothesis** **4** **(H4).** *During the COVID-19 pandemic, different content types of COVID-19 video have significant differences in the popularity level of audiences.*

The pragmatic expression of the title is the type of sentence in the video title. After the outbreak of COVID-19, affected by environmental and personal tension, people tend to share and adopt information selectively [47], where information that can attract people is more likely to be adopted. He (2018) studied Chinese texts on Internet platforms, and found that the pragmatic expression of video titles mainly includes declarative, interrogative, and exclamatory sentences [48]. Previous studies on the pragmatic expressions of titles have shown that interrogative and exclamatory titles are more popular with audiences, as they contain strong emotions that can attract the fragmented attention of audiences [49,50].

**Hypothesis** **5** **(H5).** *During the COVID-19 pandemic, different types of pragmatic expression of COVID-19 video titles have significant differences in the popularity level of audiences.*

Different from other videos, COVID-19 videos are a type of video containing medical information, such that we need to take medical information into consideration. Liu (2019) found, in a study of videos on chronic diseases, that videos with high medical information content are difficult to continuously attract patients, as patients have difficulty in understanding complex medical information, in order to make decisions and self-management; as such, they are not interested in videos with high medical information content [51]. The Medical Information and Content Index (MICI), developed by Nagpal during the Ebola epidemic [52], has been used in the research of COVID-19 videos [8,53].

**Hypothesis** **6** **(H6).** *During the COVID-19 pandemic, the lower the medical information and content index (MICI) of the video, the more popular the COVID-19 video is among audiences.*

#### 2.2.2. Source Credibility

The reliability of video information sources refers to the reliability of reference information sources in videos. It is generally believed that professional and official sources are more reliable. The reliability of information sources is an important factor affecting information adoption. In COVID-19 videos, the more reliable the information source is considered to be, the higher the quality, the stronger the audience’s perceived usefulness, and the more popular the video [54]. For the information reliability of video sources, previous studies have mostly adopted the modified DISCERN score to measure it [25,55,56]. Modified DISCERN is a 16-item scale developed by Charnock. Singh modified it to a five-item scale [57,58]. The answer to each question is yes/no, where yes is recorded as 1, and no is recorded as 0. The total score is between 0 and 5.

**Hypothesis** **7** **(H7).** *During the COVID-19 pandemic, the more reliable information sources (modified DISCERN) of COVID-19 videos are, the more popular COVID-19 videos will be.*

In addition to the credibility of the video information, trust in the uploader is an important source of trust in the uploaded video information. Previous studies have shown that audiences must first judge the credibility of the video uploader, before deciding whether to conduct further communication [59,60,61]. An audience’s trust in an uploader comes from their influence and certification on the platform [62].

**Hypothesis** **8** **(H8).** *During the COVID-19 pandemic, the greater the influence of uploaders, the more popular the videos published by them will be.*

**Hypothesis** **9** **(H9).** *During the COVID-19 pandemic, uploader’s certification types are different, and the popularity level of COVID-19 videos published by uploader is significantly different.*

#### 2.2.3. Popularity Levels

Perceived usefulness refers to the extent to which users believe that a specified system can improve their own work efficiency [32], which is ultimately manifested as information adoption. Popularity level can be used to express the results of information adoption. In the study of YouTube, popularity level was measured by views and the level of positive feedback (e.g., liking rate(likes/(likes + dislikes) × 100)) [25,27,63]. The measurement index of audience feedback is determined according to the ways that audiences can socialize on the platform. There are many different ways to socialize, including likes, shares, comments, collects, and coins. Coins are virtual currency, which allows the audience to send coins to their favorite videos, as a sign of approval.

In these social behaviors, likes, shares, collects, and coins are all types of positive feedback from the audience, while comments contain both positive and negative feedback. In order to ensure the consistency of the indicators, it is necessary to conduct sentiment analysis on the comments. There was a huge difference in the number of video comments selected in this paper, so the number of positive comments, rather than positive comment rate, was chosen as an indicator.

### 2.3. Variables Definition and Research Model

The independent variables of the research model include information quality variables (video length, title length, description length, video type, MICI, and pragmatic expression) and source credibility variables (modified DISCERN, influence, and certification type). The popularity level was indicated by the dependent variables (views, likes, shares, collects, coins, and positive comments). The definitions of the variables are provided in Table 1.

**Figure 1:**
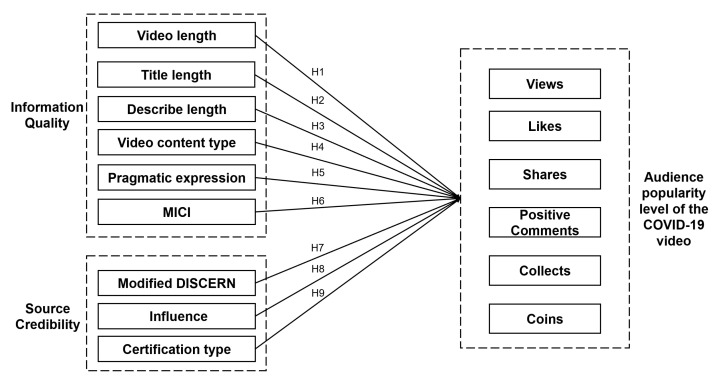
Research model.

According to the hypothesis, we can present the research model of this paper, as shown in Figure 1.

## 3. Materials and Methods

### 3.1. Data

The research data of this paper comes from a video platform in China that pays attention to originality. The strong social nature of the platform makes the platform’s audience more active, and audiences are willing to share information on the platform. Platform features and data information features can well meet the requirements of this study.

We used “COVID-19” and “COVID-19 pandemic” as search keywords, and selected data from January 2020 to February 2020, obtaining a total of 528 video data. Information of the COVID-19 videos considered in this paper included all the information needed for our research: Video features (title length, description length, video length, video link URL), uploader features (number of fans, certification type), and popularity level of the video (views, likes, comments, shares, collects, and coins).

As the search mechanism can retrieve any video with keywords in the title, tag, description, or uploader name, the search results were not necessarily relevant to COVID-19. Therefore, manual reading was used to clean the data and remove the irrelevant and missing data. A total of 251 videos highly related to COVID-19 were obtained.

### 3.2. Sentiment Analysis

Sentiment analysis is a text analysis method used to explain the emotional intensity of text [64]. A sentiment dictionary is commonly used to perform sentiment analysis. We adopted a mixed sentiment dictionary to better address the research goal of this paper. Sentiment analysis was conducted on video comments, and the comments were divided into positive, neutral, and negative, according to their emotional scores. The number of positive comments of each video was counted and recorded.

### 3.3. Subject Analysis

The subject of each video was confirmed by clustering the subject of the title and description text of the video. First, we used the jieba library for Chinese word segmentation, then processed the result after word segmentation to make the word segmentation result more accurate. Secondly, the Word2vec model was used to train the word vectors of the text after word segmentation. The clustering algorithm used was Kmeans, which has high efficiency, is simple, and has been widely used. Through the sum of squared errors (SSE) method, the best K value was determined to be 3; that is, the topics were clustered into three categories.

The results of topic clustering are shown in Table 2, where the first column is the topic class name; the second column is the part of keywords included in each category, mainly showing the words with high word frequency, while some countries and people names have been excluded; and the third column displays the actual video title. The first category involves the popularization of the relevant knowledge of COVID-19, including that relating to the virus causing COVID-19, epidemic prevention measures, and vaccines. The second category involves the praise of front-line staff. The third category involves the use of data visualization methods to analyze and display real-time epidemic data.

### 3.4. Video Coding

For the coding of video content, the modified DISCREN and MICI methods were adopted. Two researchers were invited to watch the videos independently, who checked each of the standards. If they thought the video content met the standard, they answered yes; if not, they answered no. The result of the Cohen Kappa test showed that the coding result Was reliable.

DISCREN tool is a 16-item scale developed by Charnock. Singh modified it into a 5-item scale. The answer to each question is yes/no, where “yes” is marked as 1, and “no” is marked as 0, with a total score between 0 and 5 [57,58]. The evaluation standards are shown in Table 3.

MICI is a five-item scale which evaluates from the five perspectives of prevalence, transmission, symptoms, disease diagnosis, and treatment. Each perspective includes five different scoring standards, such that the MICI scores from 0 to 25. The evaluation standards are shown in Table 4.

## 4. Results

### 4.1. Descriptive Statistical Analysis

In this paper, Stata16.0 software is used for empirical analysis.

The results of the descriptive statistical analysis of the considered 251 videos are shown in Table 5. The average score of modified DISCREN was 3.669, indicating that the COVID-19 video quality and information reliability were relatively high. Most uploaders paid more attention to video quality. The MICI score indicated that the medical information content of most COVID-19 videos was not high, as the video platform is not a professional medical platform. The number of fans of an uploader indicates influence of uploaders, with a maximum value of 6,884,873 and a minimum value of 2. However, uploaders with a large number of fans are in the minority, and there is a large gap between uploaders; therefore, the logarithm of fans of uploaders was taken as the index of influence.

From the perspective of uploader certification types (Table 6), ordinary consumers without certification (58.6%) were the main component of uploaders, and the videos uploaded by uploaders certified by institutional certification accounted for 23.9%. The analysis of video content types showed that uploaders with different certification types had posted all three different types of videos; that is, there was no difference in video content posted by uploaders with different certification types.

**Figure 2:**
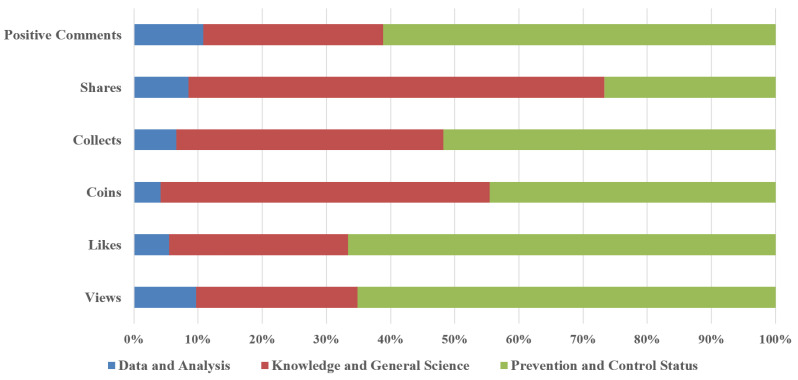
The popularity level of COVID-19 videos under different video content categories.

In terms of content types of videos (Table 7), among the 251 videos, the most (75.30%) belonged to the knowledge and general science category, while the least (11.16%) belonged to the data and analysis category. The popularity level of videos (indicated by views, likes, shares, collects, coins, and positive comments) differed greatly among the three types of videos. As shown in Figure 2, the data and analysis category videos were less popular than other types. Prevention and control status category videos obtained the most views, likes, positive comments, and collects. The knowledge and general science category videos had the highest number of coins and shares.

### 4.2. Correlation Analysis

In order to test whether there was multicollinearity, caused by strong correlations among the independent variables, we conducted a correlation analysis on the independent variables. As can be seen from Table 8, the correlation coefficients of the independent variables were all lower than 0.7.

We estimated the variance inflation factors (VIF) of the respective variables, and the results in Table 9 show that the largest VIF was 2.79—lower than the recommended threshold level of 10—indicating that multicollinearity was not a key issue in this study [65].

### 4.3. Hypothesis Testing

In this study, the popularity levels of the dependent variables (views, likes, shares, collects, coins, and positive comments) were all non-negative integers, and the variance is much larger than the mean; therefore, the negative binomial regression model was adopted for estimation. The results of hypothesis testing are shown in Table 10.

#### 4.3.1. Information Quality

H1 states that the longer the length of the COVID-19 video, the less popular it will be. Table 10 shows views (incidence rate ratio IRR = 0.997, *p* = 0.079), coins (IRR = 0.992, *p* = 0.005), likes (IRR = 0.993, *p* = 0.003), shares (IRR = 0.994, *p* = 0.015), Collects (IRR = 0.993, *p* = 0.002) and positive comments (IRR = 0.994, *p* = 0.012) were negatively correlated with length, with statistical significance. The IRR values of the six dimensions of the dependent variable indicated that, for every minute the video length increases, views decrease by 0.3%, shares and positive comments decrease by 0.6%, likes and collects decrease by 0.7%, and coins decrease by 0.8%. Thus, H1 was well-supported.

From Model 6 (IRR = 0.974, *p* = 0.003) and Model 4 (IRR = 0.982, *p* = 0.066), it can be found that, for each character increase in the title length, positive comments decrease by 2.6% and shares decreases by 1.8%; however, the *p*-values of other models were not statistically significant. Therefore, H2 was rejected.

Coins (IRR = 1.007, *p* < 0.001), likes (IRR = 1.004, *p* = 0.009), shares (IRR = 1.004, *p* = 0.007), collects (IRR = 1.006, *p* < 0.001) increased, in a statistically significant manner, as the description length increased. However, views and positive comments also showed increasing trends when the description length increased, but the results were not significant. Therefore, H3 was partially supported.

As the video content type is a categorical variable, we used the data and analysis category as the control group for negative binomial regression. Compared with data and analysis category videos, views (IRR = 2.133, *p* = 0.008), likes (IRR = 2.243, *p* = 0.007), shares (IRR = 2.187, *p* = 0.019) and collects (IRR = 2.192, *p* = 0.007) of knowledge and general science category increase significantly; views(IRR = 5.583, *p* < 0.001), likes (IRR = 5.002, *p* < 0.001), shares (IRR = 2.341, *p* = 0.048), collects (IRR = 2.287, *p* = 0.026) and positive comments (IRR = 4.357, *p* < 0.001) of Prevention and control status category also significantly increased. This demonstrates that different content types of videos are popular at different levels among audiences. Therefore, H4 was supported.

H5 states that video titles with pragmatic expression of interrogative and exclamatory sentences are more popular with audiences. However, the regression results showed that there was no significant correlation between the pragmatic expression of the title and the popularity level of the COVID-19 videos. Therefore, H5 was rejected.

Verification results for H6 showed that the popularity level of COVID-19 videos, as indicated by views (IRR = 0.908, *p* < 0.001), coins (IRR = 0.894, *p* < 0.001), likes (IRR = 0.898, *p* < 0.001), shares (IRR = 0.963, *p* = 0.041), collects (IRR = 0.932, *p* < 0.001) and positive comments (IRR = 0.888, *p* < 0.001), significantly decreased with the increase of medical information content. H6 is founded.

#### 4.3.2. Source Credibility

The hypothesis 7 verification results show that the modified DISCERN score was, indeed, suitable, with views (IRR = 1.519, *p* < 0.001), coins (IRR = 1.903, *p* < 0.001), likes (IRR = 1.584, *p* < 0.001), shares (IRR = 1.426, *p* = 0.003), collects (IRR = 1.675, *p* < 0.001) and positive comments (IRR = 1.650, *p* < 0.001) having a positive and significant effect. Taking collects as an example, the collects increased by 67.5% with every increase in the modified DISCERN score. Therefore, H7 was well-supported.

H8 states that the greater the influence an uploader has, the more popular their videos will be.The analysis of Views (IRR = 1.435, *p* < 0.001), coins (IRR = 1.683, *p* < 0.001), likes (IRR = 1.652, *p* < 0.001), shares (IRR = 1.575, *p* < 0.001), collects (IRR = 1.415, *p* < 0.001) and positive comments (IRR = 1.507, *p* < 0.001)in relation to uploader influence verified H8.

When the uploader’s certification type was that of an institutional certification, views (IRR = 0.215, *p* < 0.001), coins (IRR = 0.163, *p* < 0.001), likes (IRR = 0.214, *p* < 0.001), shares (IRR = 0.179, *p* < 0.001), collects (IRR = 0.312, *p* < 0.001) and positive comments (IRR = 0.238, *p* < 0.001) were significantly reduced. Therefore, H9 was supported.

## 5. Discussion

### 5.1. Main Research Conclusions

We adopted a combination of text analysis and empirical analysis to explore the factors influencing the popularity of COVID-19 videos in video platform. The research results showed that information quality (video length, description length, video content type, MICI) and source credibility (modified DISCERN, influence, certification type) variables had significant influences on the popularity level of COVID-19 videos (assessed in terms of views, likes, shares, coins, collects, and positive comments).

With respect to information quality, our research results indicate that the shorter the video length and the longer the description length, and the more popular the COVID-19 video. In terms of the MICI values, we found that audiences preferred COVID-19 videos with lower medical information content. In addition, we also conducted text analysis on the video content type, and found three different video types: Data and analysis, knowledge and general science, and prevention and control status. Among them, the data and analysis category is a video content type that was different from that in other past pandemics (such as Ebola), being the product of the era of big data. We found that different content types of videos attracted audiences to different degrees, especially in terms of views, likes, shares, and collects.

Considering source credibility, audiences preferred COVID-19 videos with more reliable information sources. The research in this paper found that there were no big differences in the content of videos uploaded by uploaders with different certification types, consistent with past research focused on YouTube. Compared with ordinary users without certification, audiences were not interested in videos uploaded by uploaders certificated by organizations. According to the research results on the influence of uploaders, the greater the influence of an uploader, the more popular their videos are. Previous studies on the features of uploaders, especially their influence, have mainly been conducted in online social communities, and rarely applied to video research focused on public health events. This paper innovatively included the features of uploaders into the model and verified their effects, which is of great significance.

### 5.2. Contributions of the Research

Our research has the following contributions:

First, we selected a new video as the research data source. Previous studies have mostly chosen YouTube, but their results have shown that the quality of videos on YouTube is generally low and the information sources are unreliable [55]. The research conclusion of this paper is that the uploaders in this platform pay close attention to their sources of information, and the video quality is generally high and the information sources are reliable. The data set used in this paper contained more reliable video information, thus making the research more meaningful.

Second, we innovatively considered the features of uploaders in the model. Uploader features have been extensively studied in the online health community, but rarely from the perspective of public health events. This paper, thus, has made a new attempt in this aspect.

Third, in terms of video content types, we proposed three categories of videos on social platforms through text analysis, including data and analysis, knowledge and general science, and prevention and control status. The text analysis in this paper has discovered the new video category of data and analysis, which is a product of the era of big data. The data of confirmed cases, suspected cases, and death cases in various countries and regions have been widely utilized by uploaders, in order to visually display the real-time situation of the epidemic to the audience, through use of the visualization functions of various data analysis software.

Our research has important implications for management:

In terms of the video platform, the research conclusions of this paper provide a reference for the platform to judge whether the video is likely to be popular with certain audiences. The platform can optimize the recommendation algorithm from the two aspects of information quality and source credibility, and recommend videos that may be popular to audiences, in order to improve the activity in the platform. The platform can standardize the video upload strategy, such as informing uploaders on the video upload page that the video description should be as detailed as possible, in order as to improve the overall quality of the video on the platform.

In terms of uploaders, every uploader hopes that their uploaded videos can be welcomed by more audiences. The research conclusions of this paper can help uploaders to make videos more popular, through optimizing video information quality and improving their influence on the platform. This gives uploader more angles to increase the popularity level of their videos, which is of great practical significance.

In terms of audiences, we analyzed the types of videos that audiences are attracted to. In the context of the COVID-19 pandemic, audiences hope to obtain more information through the use of video platforms. The research presented in this paper can help audiences to obtain more useful information within their fragmented time, and their anxiety can also be relieved in the process of obtaining information and socializing.

### 5.3. Limitations of the Research

There are still many shortcomings in this paper, which need to be resolved in future research. First, in terms of data set selection, only one data set was selected in this paper, which was insufficient, in terms of the ductility of the research conclusions. The main reason for choosing this platform is that the originality of the platform can ensure that the video repetition rate is low, which will not distract the audience’s attention, while its strong social level ensures active social interactions. Other known communities have a hard time comparing to it, on both counts. Second, due to the limitation of content analysis methods, some important variables (e.g., camera angle) were not used in our research model. Wang [66] has found that the camera angle of the video significantly affects the popularity level of the video, mainly by affecting the audience’s immersive experience and social participation level.

## 6. Conclusions

In this paper, we explored the factors influencing the popularity of COVID-19 videos, from the two aspects of information quality ( video length, description length, video content type, and MICI) and uploader features ( modified DISCERN, influence, and certification type). The results showed that, on the video platform, shorter video lengths, longer descriptions, more reliable video sources, and lower MICI values made the assessed COVID-19 videos more popular. Audiences are more likely to be attracted to videos in the knowledge and general science and prevention and control status categories, and are more willing to watch these videos, as well as to give their likes, shares, collects, and coins. As expected, when an uploader has higher influence, their videos are more popular with audiences. Our research conclusions can provide management suggestions for the platform, help uploaders make videos more popular with audiences, and help audiences better understand COVID-19 information, such that they can carry out prevention and control well.

## Figures and Tables

**Table 1:** Variables definition.

		Name	Description
Independent Variables	Information Quality	Video Length (VLength)	The duration of a video, in minutes
		Title Length (TLength)	The number of characters in the video title
		Description Length (DLength)	The number of characters in the video description
		Video Content Type (VCT)	For the categories of videos, 0 refers to data and analysis category, 1 refers to knowledge and general science category, and 2 refers to prevention and control status category
		Medical Information and Content Index (MICI)	The content of the video contains medical information, five scales, each dimension has five indicators, a total of 25 points
		pragmatic expression (PE)	In terms of the pragmatic expression of the title, 0 represents the declarative sentence and 1 represents the interrogative and exclamatory sentences
	Source Credibility	Modified DISCERN (mDISCERN)	It is used to measure whether the source of information in the video is true and reliable. Five scales score 5 points in total
		Certification Type (CT)	0 means no certification, 1 means personal certification, and 2 means institutional certification
		Influence	Take the logarithm of the number of fans uploader has
Dependent Variables	Popularity Level	Likes	Number of likes of the video
		Collects	Number of video collections
		Positive Comments	The number of positive reviews of the video, including each level of review
		Shares	Number of shares of the video
		Coins	Number of coins of the video
		Views	Number of views of the video

**Table 2:** Content type of COVID-19 video.

Name	Keywords	Examples
knowledge and general science category	COVID-19 epidemic, transmission, vaccine, Wuhan, wild animals, infectious diseases, science, popular science, SARS, detection	(1) The most complete explanation of COVID-19 vaccine: it is a war of science, but also a contest of great powers. (2) COVID-19 buy masks and gas mask can you gas mask personal guide
prevention and control status category	medical staff, contribution, care, public welfare, service, help, victory, frontline, go against the trend, relief station, thank you, guarantee, stick to	(1) Medical staff are caring for COVID-19 patients in a temporary hospital in Italy (2) Jianlin Ji: Psychological protection of front-line medical personnel under COVID-19
data and analysis category	data analysis, visualization, digital, real time, growth, retrospect, python, latest, cumulative, effects, design	(1) Visualization of the number of foreign COVID-19 diagnoses explosion, worst in Italy [Data visualization] (2) [Data visualization] Global outbreak, the epidemic is getting worse! Cumulative number of foreign COVID-19 diagnoses in the past two months (COVID-19, Europe, Italy, USA)

**Table 3:** Modified DISCREN evaluation standard.

Standards	Instructions
Are the goals clear and achieved?	Is the content of the video consistent with the title
Are reliable sources of information used?	The information quoted comes from reliable platforms: publications, official news organizations, etc., and the narrators are registered doctors, experts and professors, etc.
Is the information provided balanced and fair?	Is the content of the message true and fair? For example, there are no rumors
Are other sources of information listed for patient reference?	In addition to this video source also mentioned other sources of information
Are areas of uncertainty mentioned	

**Table 4:** MICI evaluation standard.

Standards	Instructions
Prevalence	Number of confirmed cases
	Number of suspected cases
	Number of deaths
	Number of seriously ill patients/proportion
	Number of countries or territories involved
Transmission	The source location of the virus
	Zoonotic transmission (i.e. contact with animals)
	Person-to-person transmission
	Incubation period
	Pathways of transmission of droplets ( wearing masks, hand washing)
Symptoms	Fever
	Upper respiratory symptoms (cough, sore throat, runny nose)
	Lower respiratory symptoms (pneumonia/shortness of breath)
	Myalgia—joint pain—lethargy
	Diarrhea
Disease Diagnosis	Mention that there is a diagnosis available
	The diagnosis was made with respiratory secretions
	Mention that PCA tests can be used for identification
	How does the diagnosis work
	Reference to diagnostic/screening criteria
Treatment	Mild symptoms can resolve by themselves
	Need to go to a hospital for treatment (mention hospitalization, ICU)
	It could be dangerous or cause death
	Disease treatment is supportive (can heal)
	Vaccination

**Table 5:** Summary statistics.

Variables	N	Mean	Median	Std.Dev.	Min	Max
PE	251	1.355	1	0.479	1	2
TLength	251	28.255	27	12.187	7	78
VLength	251	26.142	11.317	38.869	0.283	335.033
DLength	251	90.964	80	70.469	0	253
mDISCREN	251	3.669	4	1.148	1	5
MICI	251	9.606	5	9.659	0	25
Influence	251	8.814	9.428	3.699	1.099	15.745
Views	251	96,316.15	11266	452,287.1	9	620,522
Likes	251	5971.231	298	41,732.976	0	621,544
Coins	251	4277.462	49	48,670.189	0	768,000
Collects	251	1807.57	143	15,772.773	0	244,000
Comments	251	245.538	26	848.487	0	10,401
Shares	251	2076.422	76	24,897.826	0	394,000

**Table 6:** Uploader certification type distribution.

Certification Types	Frequency	Percent
No certification	147	58.6
personal certification	44	17.5
institutional certification	60	23.9
Total	251	100.0

**Table 7:** Distribution of content types of COVID-19 videos.

Video Content Type	Frequency	Percent
data and analysis category	28	11.2
knowledge and general science category	189	75.3
prevention and control status category	34	13.5
Total	251	100.0

**Table 8:** Pairwise correlations.

Variables	PE	TLength	VLength	DLength	VCT	CT	Influence	mDISCERN	MICI
PE	1.000								
TLength	0.019	1.000							
VLength	−0.277	0.063	1.000						
DLength	−0.065	0.169	0.126	1.000					
VCT	−0.002	0.009	−0.188	0.000	1.000				
CT	0.147	−0.112	−0.161	0.020	0.049	1.000			
Influence	0.279	0.028	−0.323	0.029	0.118	0.638	1.000		
mDISCERN	−0.171	−0.006	0.414	−0.005	−0.119	0.013	−0.143	1.000	
MICI	−0.345	0.015	0.593	0.006	−0.111	−0.191	−0.438	0.663	1.000

**Table 9:** Independent variable variance inflation factor.

Variable	VIF	1/VIF
MICI	2.79	0.359
Influence	2.17	0.460
mDISCERN	1.90	0.525
CT	1.78	0.563
VLength	1.65	0.606
PE	1.18	0.844
TLength	1.07	0.937
DLength	1.06	0.944
VCT	1.05	0.950
Mean VIF	1.63	

**Table 10:** Hypothesis testing.

Variables	(1) Views		(2) Coins		(3) Likes		(4) Shares		(5) Collects		(6) Comments	
	IRR	*p*	IRR	*p*	IRR	*p*	IRR	*p*	IRR	*p*	IRR	*p*
PE	0.950	0.806	0.717	0.198	1.023	0.916	0.834	0.482	0.890	0.590	1.430	0.108
TLength	0.989	0.140	0.984	0.118	0.994	0.421	0.982	0.066 (*)	1.001	0.914	0.974	0.003 (***)
VLength	0.997	0.079 (*)	0.992	0.005 (***)	0.993	0.003 (***)	0.994	0.015 (**)	0.993	0.002 (***)	0.994	0.012 (**)
DLength	1.001	0.480	1.007	<0.001 (***)	1.004	0.009 (***)	1.004	0.007 (***)	1.006	<0.001 (***)	0.002	0.103
**VCT (data and analysis category as control group)**												
knowledge and general science	2.133	0.008 (***)	1.663	0.142	2.243	0.007 (***)	2.187	0.019 (**)	2.192	0.007 (***)	1.537	0.171
Prevention and control status	5.583	<0.001 (***)	1.617	0.284	5.002	<0.001 (***)	2.341	0.048 (**)	2.287	0.026 (**)	4.357	<0.001 (***)
**CT (No certification as a control group)**												
Personal certification	0.666	0.277	1.579	0.378	0.911	0.820	0.962	0.938	1.580	0.250	0.603	0.204
institutional certification	0.215	<0.001 (***)	0.163	<0.001 (***)	0.214	<0.001 (***)	0.179	<0.001 (***)	0.312	<0.001 (***)	0.238	<0.001 (***)
Influence	1.435	<0.001 (***)	1.683	<0.001 (***)	1.652	<0.001 (***)	1.575	<0.001 (***)	1.415	<0.001 (***)	1.507	<0.001 (***)
mDISCERN	1.519	<0.001 (***)	1.903	<0.001 (***)	1.584	<0.001 (***)	1.426	0.003 (***)	1.675	<0.001 (***)	1.650	<0.001 (***)
MICI	0.908	<0.001 (***)	0.894	<0.001 (***)	0.898	<0.001 (***)	0.963	0.041 (***)	0.932	<0.001 (***)	0.888	<0.001 (***)
_cons	728.053	<0.001 (***)	1.121	0.866	3.258	0.049 (**)	2.922	0.084 (*)	2.625	0.076 (*)	1.476	0.531
PseudoR2	0.038		0.092		0.078		0.078		0.077		0.077	

*** *p* < 0.01, ** *p* < 0.05, * *p* < 0.1

## Abbreviations

The following abbreviations are used in this manuscript:

MICI	medical information and content index
